# 2D Porous Ti_3_C_2_ MXene as Anode Material for Sodium-Ion Batteries with Excellent Reaction Kinetics

**DOI:** 10.3390/molecules30051100

**Published:** 2025-02-27

**Authors:** Lan Tang, Linlin Zhang, Guohao Yin, Xin Tao, Lianghao Yu, Xiaoqing Wang, Changlong Sun, Yunyu Sun, Enhui Hong, Guangzhen Zhao, Guang Zhu

**Affiliations:** 1 School of Mechanics and Optoelectronic Physics, Anhui University of Science and Technology, Huainan 232001, China; tl19966596371@163.com (L.T.); llzhang156@163.com (L.Z.); 19155440904@163.com (G.Y.); 2 Key Laboratory of Spin Electron and Nanomaterials of Anhui Higher Education Institutes, School of Mechanical and Electronic Engineering, Suzhou University, Suzhou 234000, China; 18755467179@163.com (X.W.); happysunchanglong@126.com (C.S.); sunyunyu2008@163.com (Y.S.); 17855446020@163.com (E.H.); zhaogzgold@126.com (G.Z.); 3 Key Laboratory of Leather of Zhejiang Province, College of Chemistry and Materials Engineering, Wenzhou University, Wenzhou 325035, China; taox5802311013@163.com; 4 Materials Design Division, Department of Physics, Chemistry and Biology (IFM), Linköping University, SE-581 83 Linköping, Sweden

**Keywords:** porous MXene, SIB energy storage, ionic transport, fast-charging anodes

## Abstract

Sodium-ion batteries (SIBs) are a promising electrochemical energy storage system but face great challenges in developing fast-charging anodes. MXene-based composites are a new class of two-dimensional materials that are expected to be widely used in SIB energy storage due to their excellent electrical conductivity and stable structure. However, MXenes tend to experience interlayer stacking during preparation, which can result in poor electrochemical performance and a lower actual capacity compared to the theoretical value. In this study, the porous structure was created using a chemical oxidation method from a microscopic perspective. The porous MXene (referred to as PM) was prepared by using a low concentration of hydrogen peroxide as the pore-forming solution, which enlarged the interlayer spacing to facilitate the transport of sodium ions in the electrolyte solution. The PM with the addition of hydrogen peroxide solution achieved high-rate performance, with a capacity of 247 mAh g^−1^ at 0.1 A g^−1^ and 114 mAh g^−1^ at 10 A g^−1^. It also demonstrated long-cycle stability, with a capacity of 117 mAh g^−1^ maintained over 1000 cycles at 5 A g^−1^.

## 1. Introduction

As the surge in global electricity demand puts pressure on the energy system, renewable energy storage technologies, represented by electrochemical energy storage, have become a key research direction to achieve the goal of carbon neutrality [1,2,3]. As a new class of batteries, sodium-ion batteries (SIBs) share similar intercalation chemistry with lithium-ion batteries (LIBs) and offer advantages such as lower cost and the abundance of sodium, which makes up approximately 2.64% of the Earth’s crust [4,5,6,7]. However, the development of high-performance SIBs faces some critical issues, such as suboptimal capacity, poor conductivity, and large volume changes during ion transport [8,9,10]. In 1993, Doeff and coworkers first demonstrated the extent of Na^+^ insertion/extraction reactions into soft carbon and discussed the potential application as an anode for SIBs [11]. Subsequently, Wang et al. found that graphene materials have active sites with large interlayer distances and provide disordered structures and applied them in SIBs, but graphene materials suffer from low specific capacity [12]. Transition metal sulfides (TMSs) have also been widely investigated as anodes for SIBs due to the fact that Metal-S bonds in metal sulfides are weaker than Metal-O bonds in metal oxides, which provides a great advantage in the process of sodiation/de-sodiation [13]. Recently, Li et al. combined carbon backbone and nitrogen-doped carbon nanotubes (CNTs) with transition metal compounds (TMCs) using zeolitic imidazolium backbones as precursors to synthesize CoS_2_@CNT materials. These materials promote rapid sodium-ion penetration and electrolyte diffusion while achieving a high discharge capacity of 859.9 mAh g^−1^ at 0.1 A g^−1^ [14]. However, metal sulfides currently face issues such as large volume changes and environmental pollution during production. Therefore, it is imminent to explore and design suitable anode materials for SIBs to further improve the reversible capacity, have good structural stability, and prolong the cycle life [15,16,17,18].

Two-dimensional MXene materials have been widely investigated for applications, such as energy storage, electromagnetic interference shielding, transparent conductive electrodes, and biochemistry [9,19]. Due to their highly flexible chemical properties, MXenes have been extensively studied in various applications, including as an anode material for sodium-ion batteries. As a pseudo-capacitive sodium-ion storage material, its excellent electrical conductivity and tunable surface functional groups (-O, -OH, -F) provide numerous sodium-ion storage active centers, thereby enhancing the battery’s specific capacity. For example, Zhang et al. proposed to construct a composite consisting of MoS_2_ nanosheets, which employs hollow nitrogen–phosphorus co-doped carbon (NPC) as the supporting skeleton inside the structure and externally wraps surface functional group-rich MXene as the outer cladding, which ultimately results in a crosslinked strong composite (NPC@MoS_2_/MXene) electrode and, when applied to a sodium-ion battery, exhibits up to 453 mAh g^−1^ of reversible capacity. Huang et al. also suggested in their study that MXene with a unique composition and structure, including large specific surface area and strong mechanical strength, can be widely used in the field of energy storage [20,21]. However, the aggregation or restacking of nanosheets due to van der Waals forces impedes ion/electrolyte transport between electrodes, limiting electrolyte ion accessibility and negatively impacting electrochemical performance. In their review article, Xu et al. noted that aggregation and self-stacking phenomena in monolayer or few-layer MXene nanosheets are inherently unavoidable. These structural characteristics restrict electrolyte ion accessibility and may impede MXene’s performance optimization. To address the challenge of severe interlayer stacking, Yang et al. proposed three mitigation strategies: surface modification, heteroatom doping, and the introduction of interlayer spacers [22,23]. Related studies have found that the electrochemical energy storage of MXene can be further developed through pore structure modulation engineering [24]. However, most current research focuses on optimizing the properties of one-dimensional pore structures [25,26]. For example, some researchers have leveraged the large specific surface area and strong mechanical properties of MXene to create 3D MXene network structures with abundant macroporosity using techniques, such as 3D printing, gas foaming, sol–gel, and template methods [27,28,29,30]. Yury Gogotsi et al. recently prepared MXene nanosheets with abundant in-plane mesoporous structures through oxidative etching. The resulting pore structure and large specific surface area provide unobstructed channels for charge transfer and electrolyte storage, ensuring full contact between the electrolyte and the electrodes [31]. This paves the way for engineering studies on modulating the MXene surface pore structure. Meanwhile, Chen et al. synthesized a hierarchical flower-like MXene structure using poly (methyl methacrylate) (PMMA) microspheres as a template and anchored small Molybdenum disulfide (MoS_2_) nanosheets via a hydrothermal reaction. The resulting MXene@MoS_2_ heterostructure exhibited a unique three-dimensional (3D) porous structure that effectively addresses the volume expansion of electrode materials during Na^+^ insertion/extraction and the spontaneous stacking of MXene materials. The robust 3D structure facilitates fast and stable electron transfer, enhancing electrochemical reaction kinetics and providing a rational strategy for designing conversion-based SIB anodes [32].

Given the potential benefits of oxidation in enhancing performance, it was hypothesized that the chemical oxidation process could be more efficient and suitable for the large-scale preparation of PM structures. Moreover, electrode materials prepared using this approach are still rarely applied in SIBs. In this study, a novel, simple, and scalable chemical method involving hydrogen peroxide oxidation was employed to construct PM electrodes. We found that hydrogen peroxide oxidation can partially etch MXene nanosheets without altering their crystal structure, while increasing the interlayer spacing and creating microscopic porosity. This unique PM structure significantly enhances ion transport and enables ultra-high-rate performance. This method is also applicable to other types of MXene and may, therefore, serve as a general technique for optimizing the structure of 2D MXene nanosheets.

## 2. Results and Discussion

**Figure 1:**
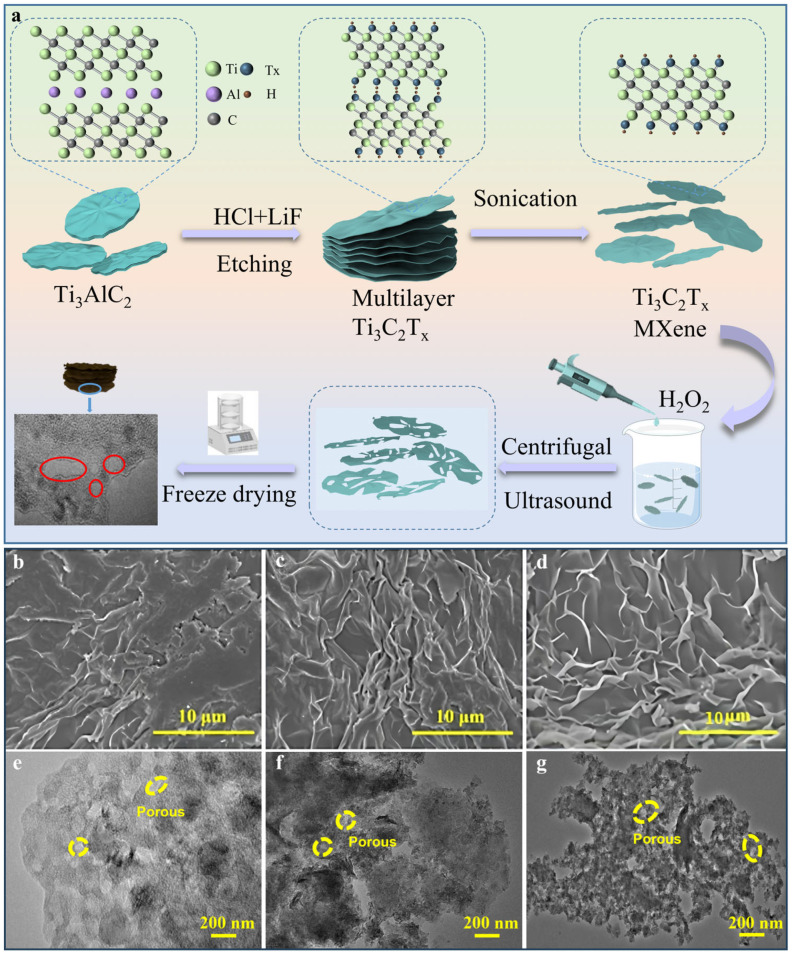
(**a**) Schematic diagram of the PM series sample preparation process; (**b**) SEM images of PM-1; (**c**) PM-2; (**d**) PM-3 at different magnifications; (**e**) TEM images of PM-1; (**f**) PM-2; (**g**) PM-3 at same magnifications.

**Figure 2:**
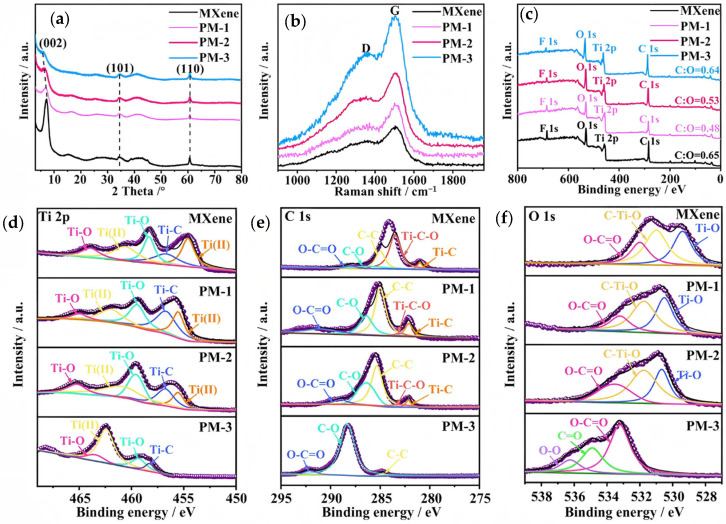
(**a**) XRD spectra; (**b**) Raman spectra; (**c**) XPS full spectrum; (**d**–**f**) XPS spectra in the Ti 2p; C 1s; and O 1s regions for all samples.

The preparation process of PM series materials is schematically shown in Figure 1a below. Different concentrations of hydrogen peroxide solution and MXene solution were mixed, and then the final PM was obtained through freeze-drying treatment. Figure 1b–d show the scanning electron microscopy (SEM) images of MXene in different contraction states after oxidation with three hydrogen peroxide concentrations. It is evident that a unique microscopic porous structure forms, with the degree of contraction increasing as the hydrogen peroxide concentration increases. This leads to more channels for ion diffusion, enhancing the material’s electrochemical performance. The transmission electron microscopy (TEM) images (Figure 1e–g) further show that the two-dimensional structure of the oxidized MXene nanosheets remains unchanged compared to the pure MXene nanosheets (Figure S1d Supplementary Materials), with only a reduction in the lateral size of individual nanosheets. This reduction in size shortens ion transport paths and results in chemical porosity, as evidenced by the formation of in-plane mesoporous structures. Yellow circles highlight the holes’ morphology for better identification. Notably, samples composed of 60 μL hydrogen peroxide solution and MXene show a higher number of pores, significantly increasing sodium transport channels and improving sodium storage performance. High-resolution TEM images (Figure S1a–c Supplementary Materials) of PM-1, PM-2, and PM-3 show multilayered crystal structures with interlayer spacings of 1.26 nm, 1.30 nm, and 1.47 nm, respectively, corresponding to the (002) plane, which is consistent with X-ray diffraction (XRD) measurements (Figure 2a).

The XRD test in Figure 2a shows that the characteristic (002) diffraction peaks of MXene shift to lower angles with increasing oxidation, with shifts of 6.88°, 6.32°, and 6.08° for PM-1, PM-2, and PM-3, respectively. This indicates a continuous expansion of the interlayer spacing and confirms the lattice spacing data discussed in the previous section [24].

The Raman spectroscopic characterization results (Figure 2b) reveals that the material exhibits typical D-peak and G-peak characteristic peaks. Among them, the G peak corresponds to the in-plane vibration mode of sp^2^ hybridized carbon atoms, reflecting the existence of graphitized ordered structure; while the D peak originates from the amorphous/defective phase vibration of sp^3^ hybridized carbon, which is closely related to the structural defects of the materials. It is noteworthy that the I_D_/I_G_ ratios of the MXene, PM-1, PM-2, and PM-3 samples increase sequentially (0.59, 0.64, 0.71, and 0.80), indicating a gradual reduction in the graphitic and ordered structures as the amount of hydrogen peroxide increases, accompanied by a significant rise in defect density. Further analysis reveals that the PM-3 sample demonstrates superior electronic conductivity and cyclic stability during sodium-ion storage. This improvement can be attributed to two factors: (1) the moderate structural defects of the C material, which create abundant edge active sites and enhance sodium-ion adsorption, and (2) the oxidative etching-induced mesoporous structure, which shortens ion diffusion paths while maintaining the continuity of the conductive network. This synergistic effect enhances both the charge transport kinetics and structural reversibility of the electrode material.

Figure 2c shows the full X-ray photoelectron spectroscopy (XPS) spectra of all the materials, confirming the presence of C, Ti, F, and O elements. Meanwhile, the proportion of each element is presented in Table S1 (Supplementary Materials). Figure 2d,e show the Ti 2p and C 1s XPS spectra. By comparing the C 1s spectra, it is observed that the Ti-C-O peak of PM-3 completely disappears, which is attributed to the increased intensity ratio of C/O. Additionally, the Ti-C peak of PM-3 decreased dramatically or even disappeared, likely due to the easy oxidation of MXene, leading to the formation of Ti-O bonds that weaken the Ti-C bond [33]. We also observe that the Ti-O bond strength of PM-3 is lower compared to the other samples. Since titanium oxide is inactive and contributes minimally to electrochemical performance, removing by-products like titanium oxide during the experimental process is crucial for achieving high capacitance and performance across multiple cycles. Figure 2f shows the O 1s XPS spectra, where the Ti-O and C-Ti-O peaks in PM-3 completely disappear, in agreement with the C 1s XPS spectra. During the etching process of MXene, which involves the oxidation of low-valence oxygen (e.g., Ti-O), the oxygen valence state is elevated, leading to the formation of C=O and O-O groups. To further analyze the specific surface area and pore size distribution of the samples, additional characterization was conducted. Figure S2a (Supplementary Materials) shows the N_2_ adsorption/desorption isotherms of all the samples, with a distinct hysteresis loop at a relative pressure of 0.5–1.0, indicating a porous structure. After treatment with hydrogen peroxide, the specific surface area of PM-3 significantly increased to 22 m^2^/g compared to the other samples. The pore size distribution curves (Figure S2b Supplementary Materials) reveal that the hydrogen peroxide-treated MXene predominantly has a mesoporous structure, which effectively provides a low-impedance pathway [34].

**Figure 3:**
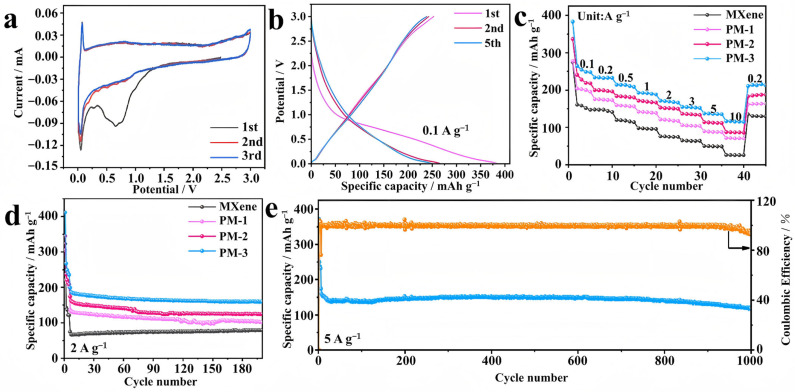
(**a**) CV curve of PM-3 electrode; (**b**) charge and discharge curve; (**c**) comparison of Rate performance of all samples; (**d**) comparison of cycles at 2 A g^−1^; (**e**) long cycle diagram of PM-3 electrode (The blue line represents the specific discharge capacity and the orange line represents the Coulomb efficiency).

The electrochemical performance of the prepared samples was tested as the negative electrode in SIBs. Figure 3a shows the cyclic voltammetry (CV) curves of the PM-3 electrode for the first three cycles at a sweep rate of 0.1 mV s^−1^. The reduction peak around 0.7 V disappears after the first cycle and does not reappear in the subsequent cycles, suggesting that this peak corresponds to an irreversible reaction. This behavior is mainly attributed to the formation of the solid electrolyte interphase (SEI) and the intercalation of sodium ions into the electrode. Figure 3b shows the constant-current charge/discharge curve of PM-3 at a current density of 0.1 A g^−1^ during the initial cycle (first five cycles). The initial capacity reaches 486 mAh g^−1^, but it decays to 233 mAh g^−1^ during subsequent cycles, resulting in an initial coulombic efficiency (ICE) of 47.9%. The rapid capacity decay is likely due to the irreversible reaction of the active material and the formation of the SEI layer. Notably, the charge and discharge curves of the second and fifth cycles are nearly identical, indicating that the charge/discharge reactions are highly reversible.

Figure 3c compares the performance of MXene, PM-1, PM-2, and PM-3 in terms of rate capability at different current densities. The PM-3 electrode exhibits the best rate capability, achieving a capacity of 247 mAh g^−1^ at 0.1 A g^−1^. Even at a high current density of 10 A g^−1^, the PM-3 electrode retains a capacity of 114 mAh g^−1^, demonstrating its excellent rate capability. This can be attributed to its porous structure, which facilitates ion transport. Notably, after 200 cycles at 2 A g^−1^, the PM-3 electrode maintains a capacity of 159 mAh g^−1^, outperforming the other samples and showing superior cycling stability (Figure 3d). This performance is due to the enlarged interlayer spacing and structural stability. The coulombic efficiency (CE) of the PM-3 electrode quickly reaches nearly 99% after several cycles, indicating good reversibility. Furthermore, after 1000 cycles at 5 A g^−1^, the capacity stabilizes at 117 mAh g^−1^, demonstrating excellent long-term cycling stability (Figure 3e). The outstanding electrochemical performance of PM-3 is attributed to its porous structure, which alleviates negative effects such as volume expansion during energy storage. Thus, the PM-3 electrode material, with its higher capacity and better cycling stability, holds significant potential for energy storage applications (Table S4 Supplementary Materials).

**Figure 4:**
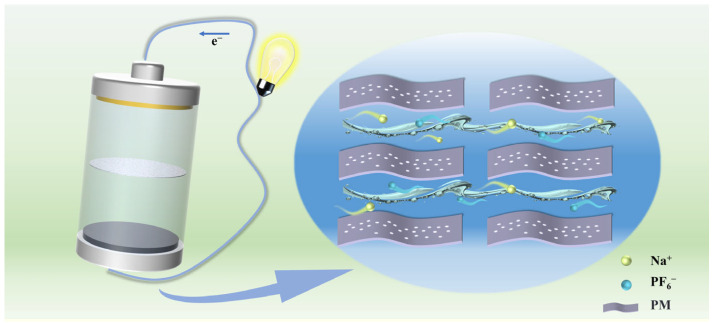
Schematic representation of the ion transport rate of PM-3.

**Figure 5:**
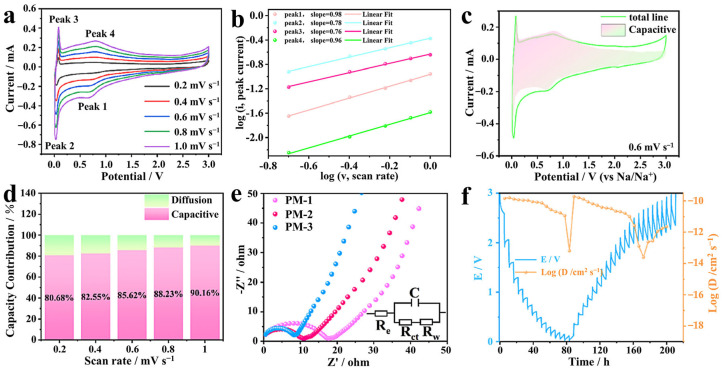
The PM-3 electrode (**a**) CV curves; (**b**) current and sweep velocity plots of the peaks; (**c**) pseudocapacitance contribution plots (0.6 mV s^−1^); (**d**) capacitance contribution plots at different sweep speeds; (**e**) comparison of impedance data for PM-1, PM-2 and PM-3; (**f**) GITT and Na^+^ diffusion coefficient curves of the PM-3 electrode.

The improved cycling and rate performance of PM-3 samples prompted us to further investigate their charge storage mechanisms. Figure 4 shows a schematic diagram of ion transport in PM-3 samples. Using CV measurements at different scan rates, we analyzed the capacitance characteristics of all the samples (Figure 5a, Figure S3a, S4a and S5a Supplementary Materials). The capacitance contribution to the total capacity was qualitatively evaluated by the relationship between scan rate (*v*) and the measured current (*i*), which enables us to quantify PM-3’s contribution to the total capacity using Equations (1) and (2). The equations are as follows [35,36,37]:

(1) $$\mathrm{i}=av^{b}$$

(2) $$\log\left(\mathrm{i}\right)=b\log\left(v\right)+\log\left(a\right)$$

where the b-value indicates the type of electrochemical behavior, determined by the slope in the plot of log(*i*) versus log(*v*). It serves as a typical indicator of charge storage kinetics. For diffusion-controlled processes, the b-value is close to 0.5, while for processes dominated by capacitive behavior (non-diffusion-controlled), the b-value is close to 1. For the PM-3 electrodes, the calculated b-values corresponding to the peaks in Figure 5b are 0.98, 0.78, 0.76, and 0.96, respectively. These values are all close to 1, indicating that the PM-3 electrodes exhibit good capacitive behavior. The calculated b-values for MXene, PM-1, and PM-2 electrodes are 0.68 and 0.54, 0.79 and 0.72, and 0.74 and 0.61, respectively (Figure S3b, S4b, and S5b Supplementary Materials). These values also suggest that the electrochemical behavior of these electrodes is dominated by capacitive processes during sodium storage, indicating that the contribution of non-diffusion-controlled capacitance increases with the development of the porous structure [38].

In order to further quantitatively distinguish the contribution of the non-diffusive part to the total current at a given scan rate, it is expressed computationally using Equation (3) [39,40]:

(3) $$\mathrm{i}=k_{1}v+k_{2}v^{\frac{1}{2}}$$

where $k_{1}v$ represents the non-diffusive contribution and $k_{2}v^{\frac{1}{2}}\;$ represents the diffusion-controlled sodium-ion intercalation effect. The $k_{1}\;$ and $k_{2}\;$ values are obtained by calculating the relationship between *i* and *v*. The non-diffusive contributions are shown as shaded portions in the CV curves, and the calculated capacitive contributions for PM-3, MXene, PM-1, and PM-2 electrodes are 85.62%, 79.87%, 82.26%, and 85.69% at 0.6 mV s^−1^ (Figure 5c, Figure S3c, S4c and S5c Supplementary Materials), and they gradually increased to 90.16%, 84.13%, 86.28%, and 89.54% at 1 mV s^−1^ (Figure 5d, Figure S3d, S4d and S5d Supplementary Materials). This indicates that the capacitive contribution increases with the scanning rate. The results demonstrate that PM-3 outperforms the other materials, with its porous structure offering a large specific surface area and more active sites for surface redox reactions, which also contributes to its excellent multiplicative performance at high current densities.

Figure 5e shows the impedance spectrum of the PM series. In this spectrum, there is a semicircle and an inclined line in the low-frequency region. The smaller the diameter of the semicircle, the smaller the charge transfer resistance (R_ct_). The inclined line in the low-frequency region represents the ion diffusion resistance (R_w_). The greater the slope of this slash, the faster the diffusion rate [41]. The R_ct_ of PM-3 is significantly lower compared to MXene, PM-1, and PM-2, suggesting a faster charge transfer rate. Additionally, a more developed pore structure results in higher R_w_ value, providing ample channels for electrolyte penetration. Furthermore, as shown in Figure 5f and Figure S6a–c, we evaluated the kinetics by performing the galvanostatic intermittent titration technique (GITT) test and calculating the sodium ion diffusion coefficients for all samples using Equation (4) [42,43], which is given below:

(4) $$D^{GITT}=\frac{4}{\pi \tau }{\left(\frac{m_{B}V_{M}}{M_{B}S}\right)}^{2}{\left(\frac{∆E_{s}}{∆E_{t}}\right)}^{2\;\;}$$

In the equation, τ denotes the constant current pulse duration, while *m_B_*, *M_B_*, and *V_M_* represent the mass, molar mass, and molar volume of the active substance, respectively. *S* is the total contact area between the electrode material and the electrolyte (for simplicity, we adopted a geometric surface area with a value of 1.13 cm^2^), and Δ*E_s_* and Δ*E_t_* are the steady-state voltage change minus the IR voltage drop and the total transient change in cell voltage during a single titration, respectively [44]. The diffusion coefficients of all three PM series electrodes are higher than those of the pure MXene electrodes, with the PM-3 electrode showing the best performance. These results suggest that the porous structure enhances electrolyte diffusion and accelerates sodium-ion diffusion, which reduces resistance and ultimately improves both capacity and multiplicity performance.

**Figure 6:**
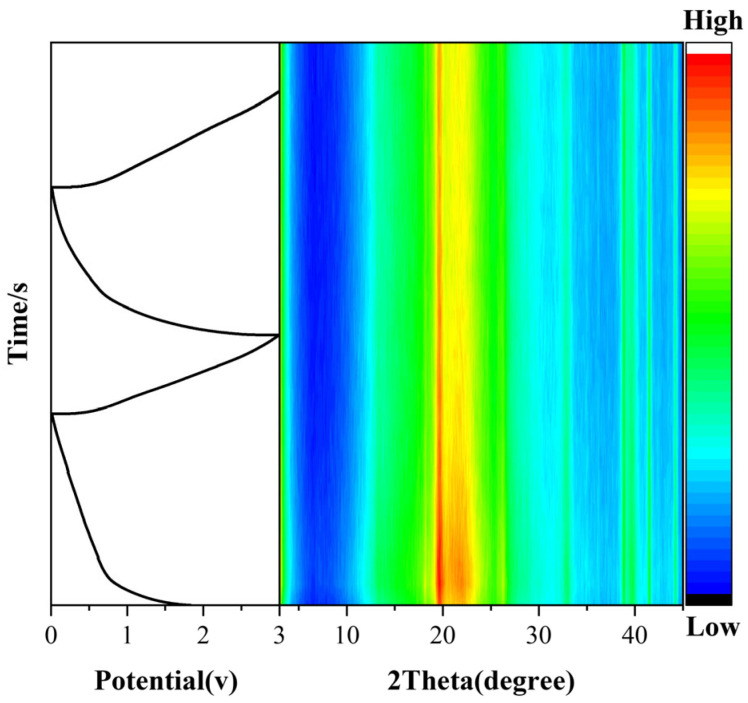
In situ XRD data for PM-3.

In situ XRD measurements were performed at different discharge and charge stages of the cell to explore the changes in the microstructure of the electrode material throughout the charge–discharge cycle. As shown in Figure 6, the characteristic peaks of the (002) crystal face were obviously weakened or even disappeared during the charging and discharging process, which may be due to the fact that hydrogen peroxide as an oxidizing agent may etch away part of the MXene layer, leading to the partial collapse of the lamellar structure, and the spacing of the treated MXene layers increased; thus, the intensity of the (002) peaks was reduced, and this conclusion is also well represented in Figure 2a and Figure S1a–c, respectively. Two strong peaks can be observed in the range of 18.5° to 27°, a phenomenon attributed to the more ordered stacking of the PM layers compared to the original MXene layers. Additionally, the (101) crystallographic diffraction peaks are shifted to a lower angle (33°–34°) during electrochemical cycling, indicating the insertion of Na^+^ between the Ti_3_C_2_T_x_ layers and the increase in interlayer spacing, resulting in the formation of Na_y_Ti_3_C_2_T_x_. The reaction mechanism is shown in Equation (5). No new diffraction peaks were observed throughout the charging and discharging process. In summary, the Na^+^ storage mechanism of PM follows a typical intercalation-conversion mechanism, consistent with the CV diagram shown earlier [45,46].

(5) $${Ti}_{3}C_{2}T_{x}+y{Na}^{+}+ye^{-}={Na}_{y}{Ti}_{3}C_{2}T_{x}$$

## 3. Experimental Section

### 3.1. Preparation of Ti_3_C_2_T_x_ MXene Nanosheets

Ti_3_C_2_T_x_ MXene was prepared by etching Ti_3_AlC_2_ MAX (11 technology co., LTD) with LiF and HCl. Firstly, 2 g of LiF was taken with a medicine spoon as well as HCl (12 M, 40 mL) solution with a pipette gun in a reactor and stirred for 30 min to achieve sufficient mixing. Subsequently, 2 g of Ti_3_AlC_2_ MAX was slowly added to the mixed solution, and the water bath was heated for the etching process (35 °C, 24 h). The mixture was then washed several times with deionized water until the supernatant became neutral. The obtained multilayered MXene was homogeneously dispersed in 200 mL of deionized water with stirring for 10 min, then treated with inert gas to prevent oxidation during subsequent sonication. Finally, sonication was carried out for 60 min in an ice-water bath (0 degrees centigrade).

### 3.2. Preparation of PM

H_2_O_2_ (30%) (15 µL) was first added to the MXene solution with continuous stirring at room temperature for 1 h. Subsequently, the by-product TiO_2_ was removed via centrifugation several times with HCl solution and deionized water. The precipitate was then re-dispersed into deionized water (25 mL) and sonicated for 5 min to obtain the PM solution. Finally, deionized water (25 mL) was added to the PM solution (30 mg) and then sonicated for 5–10 min to evenly disperse both. Next, the target sample was obtained after freeze-drying. Different amounts of H_2_O_2_ (15 µL, 45 µL and 60 µL) were added by analogy, and the PM materials formed were labeled as PM-1, PM-2 and PM-3, respectively.

### 3.3. Material Characterization

The morphological characteristics of the materials were tested using scanning electron microscopy (SEM, Nova Nanosem 200 system, 10 kV, Hillsboro, OR, USA). In order to show the morphology of the samples in more detail, transmission electron microscopy tests were performed (TEM, JEOL-2100, accelerating voltage 200 kV, Geo Lu (Beijing) Science and Technology Co. Ltd., Beijing, China). Monochromatic X-ray photoelectron spectroscopy was performed at 22.4 W Al Kα radiation (1486.7 eV) (XPS, Kratos Axis Ultra DLD, Kratos, Manchester, UK). The acquired XPS data were processed using the XPS Peak software (XPS Peak Fit V4.1). The crystal structure and phase composition of the samples were analyzed using X-ray diffraction (XRD, Bruker D8 Advance with Cu Kα radioactive source, Karlsruhe, Germany). The XRD measurements were conducted in an angle range of 3° to 80°. Raman spectroscopy was used to further determine the molecular structure and vibrational information of the material. The excitation wavelength used was 532 nm, and the test range was 100–4000 cm^−1^. Nitrogen adsorption/desorption testing was performed to analyze the specific surface area and pore structure of the material. The Brunauer–Emmett–Teller (BET) method was used to derive the specific surface area.

### 3.4. Electrochemical Measurements

According to the ratio of 7:2:1, the active material, conductive carbon, and binder Polyvinylidene fluoride (PVDF) were mixed and stirred in N-methyl-2-pyrrolidinone (NMP) solvent for 30 min to obtain a homogeneous slurry, and then the slurry was uniformly coated on the copper foil. Then, the obtained slurry was evenly coated on the copper foil and dried in a vacuum drying oven overnight (80 °C). The mass of the active substance was weighed to be approximately 1.0 mg, and, finally, it was dried in a vacuum drying oven for 4 h and then put into a glove box to prepare for the assembly of the cell. The battery type was a CR-2025 button cell, and the battery was assembled in a glove box filled with argon gas (H_2_O < 0.1 ppm, O_2_ < 0.1 ppm). The electrolyte was a solution of 1 M NaPF_6_ in DME = 100 Vol%. The assembled cells were left to stand for 10 h, and then subsequent performance tests were conducted at room temperature. The cyclic voltammetry test equipment was a Chi660e electrochemical workstation with a test window of 0.01–3.0 V. The scanning rates were set to 0.1, 0.2, 0.4, 0.6, 0.8 and 1 mV s^−1^. The constant current charge/discharge test equipment was the New Ware Battery Test System (the system environment was maintained at a constant temperature of 25 °C), with a voltage range of 0.01–3.0 V. After the battery is left to stand for 10 h, it will be activated by a low-current-density activation operation. The test frequency range used for electrochemical impedance spectroscopy is from 0.01 Hz to 1000 kHz. The constant current intermittent titration test voltage range is 0.01–3 V, and the current density used is 0.1 A g^−1^. For the galvanostatic intermittent titration technique (GITT) tests, the cell is charged and discharged at a current density of 0.1 A g^−1^, with a constant current charge and discharge time of 10 min and a relaxation time of 90 min. The linear fit correlation R^2^ for Figure 5b, Figure S3b, S4b and S5b is shown in Tables S2 and S3 (Supplementary Materials). The in situ XRD cell consists of an electrode material, a beryllium window, and a cut sodium sheet. X-rays can penetrate the beryllium window to detect the evolution of the phase structure of the sample. The test angle ranges from 5° ≤ 2θ ≤ 45°. And, combined with the galvanostatic charging/discharging (GCD) test, the in situ cell was connected to the electrochemical workstation via electrode wires after assembly. The GCD test was performed at 0.05 A g^−1^. The whole process ended when the GCD test was completed.

## 4. Conclusions

In conclusion, we developed a simple method for oxidizing MXene with hydrogen peroxide, which effectively addresses the issue of MXene aggregation. The PM-3 electrode, with its relatively large specific surface area (22 m^2^/g), demonstrates excellent performance: a capacity of 159 mAh g^−1^ after 200 cycles at 2 A g^−1^, impressive rate capability (247 mAh g^−1^ at 0.1 A g^−1^ and 114 mAh g^−1^ at 10 A g^−1^), and remarkable long-term cycling stability (capacity stabilized at 117 mAh g^−1^ after 1000 cycles at 5 A g^−1^). This result provides new insights into the application of MXene materials in SIBs, which is particularly helpful for the development of high-performance sodium-ion battery anodes. However, there are still many possibilities to improve and expand the methodology of this study. In a subsequent study, we plan to investigate the specific reaction mechanism of MXene oxidation by hydrogen peroxide and clarify the influence of each reaction condition on the morphology and properties of MXene, so as to achieve more precise control of the properties of MXene materials. At the same time, we will try to combine this method with other modification techniques, such as compositing porous MXene with high-capacity materials (e.g., Sn, P) to construct a synergistic system of “conductive skeleton-active sites”, which will further enhance the energy density of the full battery and provide strong support for the commercialization of high-performance sodium-ion batteries.

## Data Availability

Data are contained within the article and Supplementary Materials.

## Supplementary Materials

The following supporting information can be downloaded at: https://www.mdpi.com/article/10.3390/molecules30051100/s1, Figure S1. HRTEM images of (a) PM-1; (b) PM-2; and (c) PM-3; (d) TEM images of MXene. Figure S2. (a) N_2_ suction/desorption isotherms; (b) pore size distribution curves for all samples. Figure S3. The MXene electrode (a) CV curves; (b) current and sweep velocity plots of the peak; (c) pseudocapacitance contribution plots (0.6 mV s^−1^); and (d) capacitance contribution plot at different sweep speeds. Figure S4. The PM-1 electrode (a) CV curves; (b) the current value of the peak vs. the sweep speed; (c) the pseudocapacitance contribution plot (0.6 mV s^−1^); and (d) the capacitance contribution rate plot at different sweep speeds. Figure S5. The PM-2 electrode (a) CV curves; (b) the current value of the peak vs. the sweep speed; (c) the pseudocapacitance contribution diagram (0.6 mV s^−1^); and (d) the comparison of capacitance contribution at different sweep speeds. Figure S6. GITT and Na^+^ diffusion coefficient curve of the (a) MXene; (b) PM-1 and (c) PM-2 electrode. Table S1. XPS quantitative analysis of the ratio of C, F, O, and Ti on the surface of MXene and PM-3. Table S2. Peak current and sweep speed fit similarity R^2^ of PM-3 electrode. Table S3. Peak current and sweep speed fit similarity R^2^ of MXene, PM-1, PM-2 electrode. Table S4. The cycling performance and capacity decay from materials reported recently in literature [47,48,49,50,51,52,53,54,55,56,57,58].
