# Supplementary material for: 2D Porous Ti3C2 MXene as Anode Material for Sodium-Ion Batteries with Excellent Reaction Kinetics
Source: Molecules. 2025 Feb 27;30(5):1100. doi: 10.3390/molecules30051100 (PMC11901979; doi:10.3390/molecules30051100)
Supplement: Supplementary file 1 [file molecules-30-01100-s001.zip › molecules-3460589-supplementary.pdf]

2D Porous Ti3C2 MXene as Anode Material for Sodium-Ion Batteries with Excellent Reaction Kinetics

Lan Tang 1,2, Linlin Zhang 1,2, Guohao Yin 1,2, Xin Tao 3, Lianghao Yu 2,4,*, Xiaoqing Wang 2, Changlong Sun 2, Yunyu Sun 2, Enhui Hong 2, Guangzhen Zhao 2 and Guang Zhu 2,*

1 School of Mechanics and Optoelectronic Physics, Anhui University of Science and Technology, Huainan 232001, China
2 Key Laboratory of Spin Electron and Nanomaterials of Anhui Higher Education Institutes, School of Mechanical and Electronic Engineering, Suzhou University, Suzhou 234000, China
3 Key Laboratory of Leather of Zhejiang Province, College of Chemistry and Materials Engineering, Wenzhou University, Wenzhou 325035, China
4 Materials Design Division, Department of Physics, Chemistry and Biology (IFM), Linköping University, SE-581 83 Linköping, Sweden
*Correspondence: lianghao.yu@liu.se (L.Y.); guangzhu@ahszu.edu.cn (G.Z.)

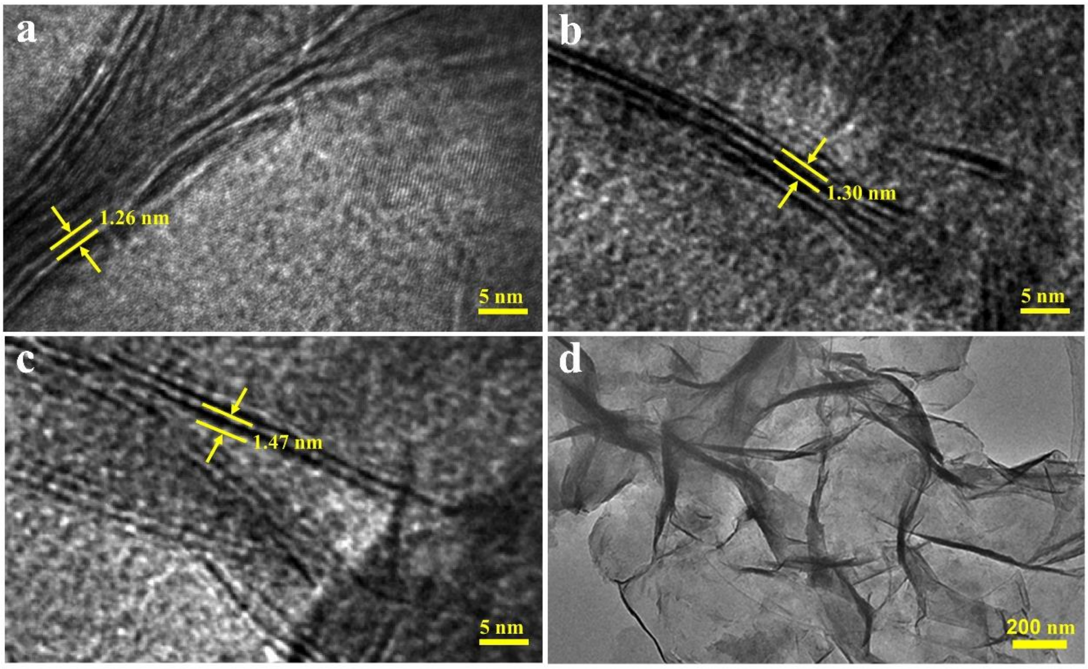

Figure S1. HRTEM images of (a)PM-1; (b) PM-2; and (c) PM-3; (d) TEM images of MXene.

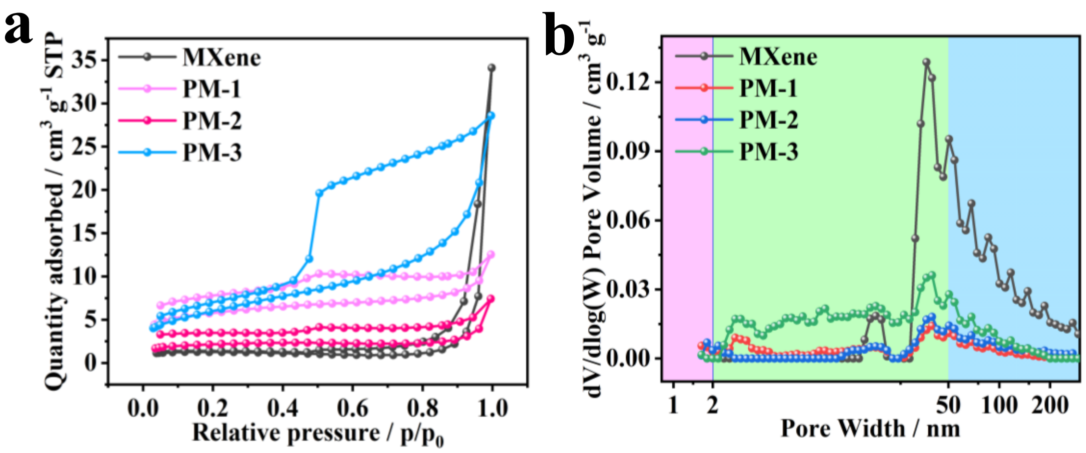

Figure S2. (a) N_2 suction/desorption isotherms; (b) pore size distribution curves for all samples.

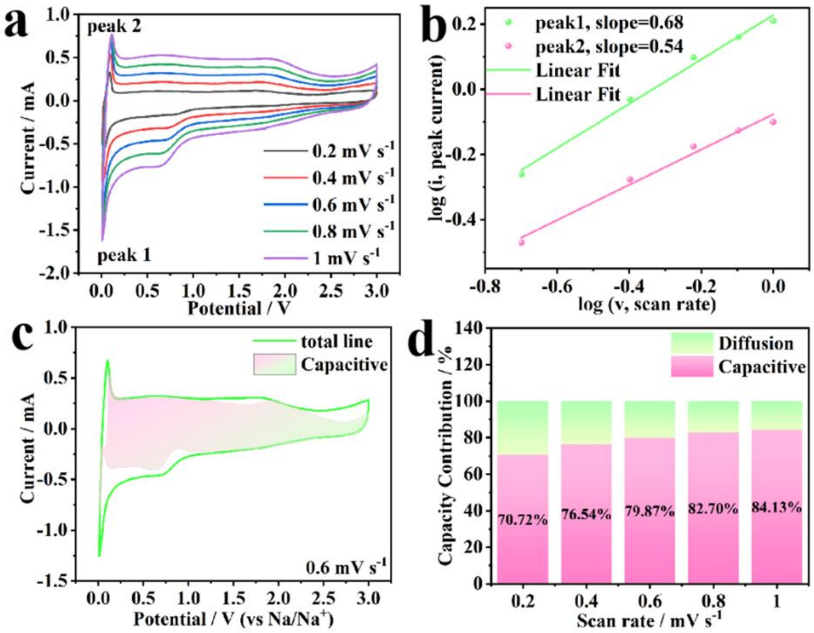

Figure S3. The MXene electrode (a) CV curves; (b) current and sweep velocity plots of the peak; (c) pseudocapacitance contribution plots (0.6 mV s^{-1}); and (d) capacitance contribution plot at different sweep speeds.

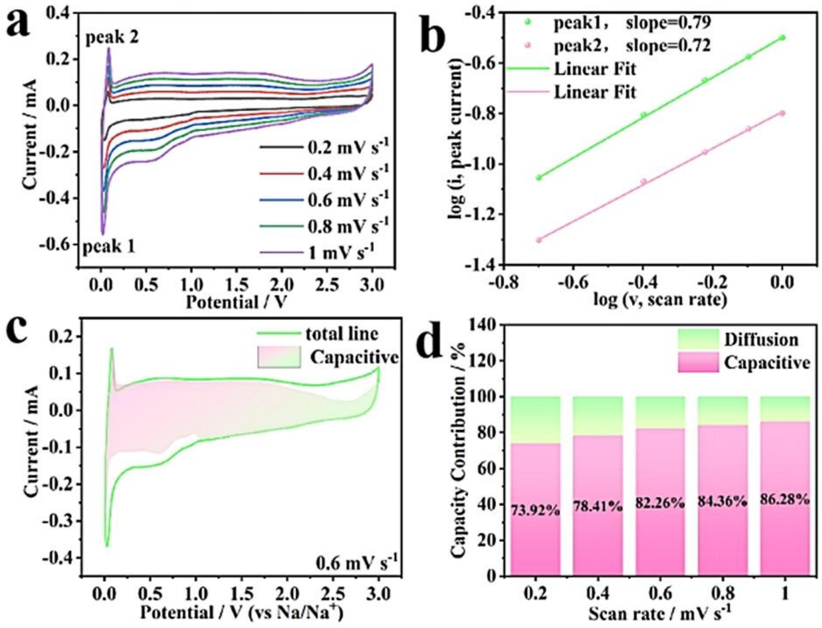

Figure S4. The PM-1 electrode (a) CV curves; (b) the current value of the peak vs. the sweep speed; (c) the pseudocapacitance contribution plot (0.6 mV s^{-1}); and (d) the capacitance contribution rate plot at different sweep speeds.

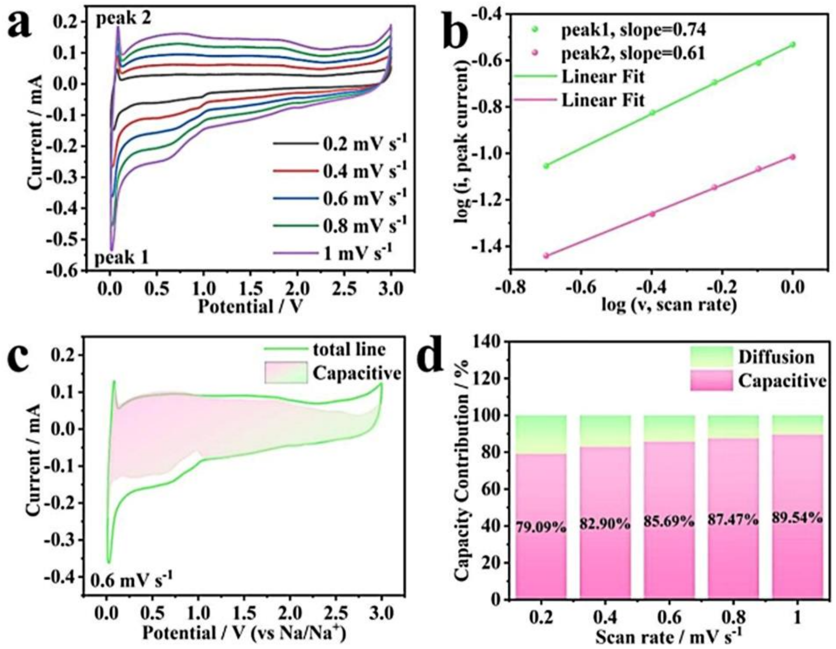

Figure S5. The PM-2 electrode (a) CV curves; (b) the current value of the peak vs. the

sweep speed; (c) the pseudocapacitance contribution diagram (0.6 mV s^{-1}); and (d) the comparison of capacitance contribution at different sweep speeds.

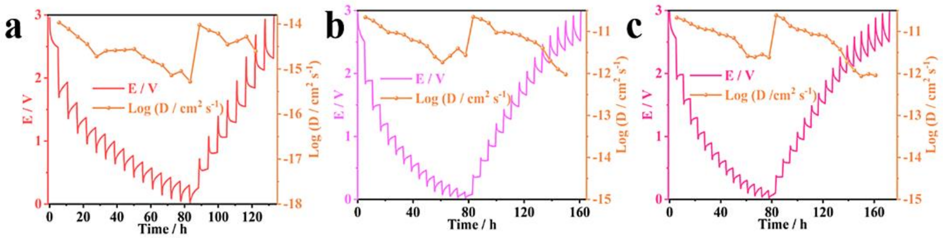

Figure S6. GITT and Na^+ diffusion coefficient curve of the (a) MXene; (b) PM-1 and (c) PM-2 electrode.

Table S1. XPS quantitative analysis of the ratio of C, F, O, and Ti on the surface of MXene and PM-3.

XPS	C	F	O	Ti
**MXene**	36.78%	17.36%	17.21%	28.65%
**PM-3**	37.56%	12.18%	23.57%	26.69%

Table S2. Peak current and sweep speed fit similarity R^2 of PM-3 electrode.

PM-3	Peak1	Peak2	Peak3	Peak4
R^2	0.999	0.999	0.997	0.998

Table S3. Peak current and sweep speed fit similarity R^2 of MXene、PM-1、PM-2 electrode.

MXene	Peak1	Peak2	PM-1	Peak1	Peak2	PM-2	Peak1	Peak2
R^2	0.992	0.983	R^2	0.999	0.999	R^2	0.999	0.999

Table S4. The cycling performance and capacity decay from materials reported recently in literature.

Material	Current (A g^{-1})	Cap. (mAh g^{-1})	Cycle	Ref.
V_2C MXene	2	54	1800	**[1]**
$Na_2Ti_3O_7$@C	2	119	200	**[2]**
Sulfur-decorated Ti_3C_2 MXenes	2	135	1000	**[3]**
DPMM	0.1	160	1000	**[4]**
f-$Ti_3C_2T_x$_DMSO	1	76	1500	**[5]**
$Ti_3C_2T_x$/PDDA-GO	5	65	1000	**[6]**
TBC/$Ti_3C_2T_x$	1	0.1	100	**[7]**
NTO/Ti_3C_2	2	82	1900	**[8]**
K-PMM	0.1	188	1500	**[9]**
Mo_2C MXene	0.01	50	140	**[10]**
T-MXene@C	1	139.5	3000	**[11]**
Ti_3CNT_x	0.1	110	200	**[12]**
PAQS@MXene	5	57	9000	**[13]**
PM-3	2<br>5	159<br>117	200<br>1000	**This work**

References

1. Zhang, W.; Peng, J.; Hua, W.; Liu, Y.; Wang, J.; Liang, Y.; Lai, W.; Jiang, Y.; Huang, Y.; Zhang, W.; Yang, H.; Yang, Y.; Li, L.; Liu, Z.; Wang, L.; Chou, S. L. Architecting Amorphous Vanadium Oxide/MXene Nanohybrid via Tunable Anodic Oxidation for High-Performance Sodium-Ion Batteries. *Advanced Energy Materials.* **2021**, 11, 2100757.
2. Zhong, W.; Tao, M.; Tang, W.; Gao, W.; Yang, T.; Zhang, Y.; Zhan, R.; Bao, S.-J.; Xu, M. MXene-derivative pompon-like $Na_2Ti_3O_7$@C anode material for advanced sodium ion batteries. *Chemical Engineering Journal.* **2019**, 378, 122209.
3. Sun, S.; Xie, Z.; Yan, Y.; Wu, S. Hybrid energy storage mechanisms for sulfur-decorated Ti_3C_2 MXene anode material for high-rate and long-life sodium-ion batteries. *Chemical Engineering Journal.* **2019**, 366, 460-467.
4. Niu, B.; Zhang, Y.; Long, Y.; Zhao, J.; Li, Q.; Zhang, B.; Tao, Y.; Yang, Q.-H. Concentrated Laminate Structure in Dense MXene Monoliths Promises High-Capacity Sodium Storage. *Small Structures.* **2023**, 4, 2300143.
5. Wu, Y.; Nie, P.; Wang, J.; Dou, H.; Zhang, X. Few-Layer MXenes Delaminated via High-Energy Mechanical Milling for Enhanced Sodium-Ion Batteries Performance. *ACS Appl Mater Interfaces.* **2017**, 9, 39610-39617.
6. Liu, J.; Chang, Y.; Guo, H.; Cao, D.; Sun, K.; Wang, T.; Liu, D.; Fu, Y.; Liu, J.; He, D. Self-assembled $Ti_3C_2T_x$ /poly(diallyldimethylammonium chloride)-graphene oxide multilayers with large layer spacing for high capacity sodium-ion batteries. *Journal of Power Sources.* **2024**, 624, 235492.
7. Li, J.; Hao, J.; Wang, R.; Yuan, Q.; Wang, T.; Pan, L.; Li, J.; Wang, C. Ultra-stable cycling of organic carboxylate molecule hydrogen bonded with inorganic $Ti_3C_2T_x$ MXene with improved redox kinetics for sodium-ion batteries. *Battery Energy.* **2023**, 3, 20230033.
8. Sun, X.; Tan, K.; Liu, Y.; Zhang, J.; Hou, L.; Yuan, C. In-situ growth of hybrid $NaTi_8O_{13}/NaTiO_2$ nanoribbons on layered MXene Ti_3C_2 as a competitive anode for high-performance sodium-ion batteries. *Chinese Chemical Letters.* **2020**, 31, 2254-2258.
9. Zhao, J.; Li, Q.; Shang, T.; Wang, F.; Zhang, J.; Geng, C.; Wu, Z.; Deng, Y.; Zhang, W.; Tao, Y.; Yang, Q.-H. Porous MXene monoliths with locally laminated structure for enhanced pseudo-capacitance and fast sodium-ion storage. *Nano Energy.* **2021**, 86, 106091.
10. Mei, J.; Ayoko, G. A.; Hu, C.; Bell, J. M.; Sun, Z. Two-dimensional fluorine-free mesoporous Mo_2C MXene via UV-induced selective etching of Mo_2Ga_2C for energy storage. *Sustainable Materials and Technologies.* **2020**, 25. e00156.
11. Zhang, P.; Soomro, R. A.; Guan, Z.; Sun, N.; Xu, B. 3D carbon-coated MXene architectures with high and ultrafast lithium/sodium-ion storage. *Energy Storage Materials* **2020**, 29, 163-171.
12. Zhang, W.; Liu, S.; Chen, J.; Hu, F.; Wang, X.; Huang, H.; Yao, M. Exploring the Potentials of $Ti_3C_iN_{2-i}T_x$ (i = 0, 1, 2)-MXene for Anode Materials of High-Performance Sodium-Ion Batteries. *ACS Appl Mater Interfaces.* **2021**, 13, 22341-22350.
13. Gao, Y.; Xue, P.; Ji, L.; Pan, X.; Chen, L.; Guo, W.; Tang, M.; Wang, C.; Wang, Z. Interfacial Self-assembly of Organics/MXene Hybrid Cathodes Toward High-Rate-Performance Sodium Ion Batteries. *ACS Appl Mater Interfaces.* **2022**, 14, 8036-8047.
